# Supplementary material for: Preparation and Characterization of Salt-Mediated Injectable Thermosensitive Chitosan/Pectin Hydrogels for Cell Embedding and Culturing
Source: Polymers (Basel). 2021 Aug 10;13(16):2674. doi: 10.3390/polym13162674 (PMC8398595; doi:10.3390/polym13162674)
Supplement: Supplementary file 1 [file polymers-13-02674-s001.zip › Supplementary Materials.pdf]

Supporting Materials

Preparation and characterization of salt-mediated injectable thermosensitive chitosan/pectin hydrogels for cell-embedding and culturing

G. Morello [1,2,†], A. Polini [2,†,*], F. Scalera [2], R. Rizzo [2], G. Gigli [1,2], and F. Gervaso [2,*]

[1] Dipartimento di Matematica e Fisica E. De Giorgi, University of Salento, Campus Ecotekne, via Monteroni, 73100 Lecce, Italy; giulia.morello@unisalento.it; giuseppe.gigli@unisalento.it
[2] CNR NANOTEC – Institute of Nanotechnology c/o Campus Ecotekne, via Monteroni, 73100 Lecce, Italy; alessandro.polini@nanotec.cnr.it; francesca.scalera@nanotec.cnr.it; riccardo.rizzo@nanotec.cnr.it; francesca.gervaso@nanotec.cnr.it
† These authors equally contributed to this study.
* Correspondence: alessandro.polini@nanotec.cnr.it (AP), francesca.gervaso@nanotec.cnr.it (FG); Tel.: +39 0832 319305

Table S1. Hydrogel formulations based of CH+PEC with different GAs, with and without addition of complete DMEM.

**Hydrogel compositions**	
βGP	2.5 mL (Ch/Pec) + 0.5 mL βGP (0.04 M)
PB	2.5 mL (Ch/Pec) + 0.5 mL PB (0.04 M)
SHC	2.5 mL (Ch/Pec) + 0.5 mL SHC (0.04 M)
βGP-DMEM	2.5 mL (Ch/Pec) + 0.5 mL βGP (0.04 M) + 0.5 mL DMEM
PB-DMEM	2.5 mL (Ch/Pec) + 0.5 mL PB (0.04 M) + 0.5 mL DMEM
SHC-DMEM	2.5 mL (Ch/Pec) + 0.5 mL SHC (0.04 M) + 0.5 mL DMEM

Table S2. Schematic representation of three hydrogel formulations (βGP-PB-SHC) inversion tube test and injection test through G23 needle at r.t.

**Inversion Tube Test**	**βGP**	**PB**	**SHC**
t=0 r.t.	No flow	No Flow	No Flow
t=2 h 37°C	/	/	/
Injectability (r.t.)	Injectable	Injectable	Injectable

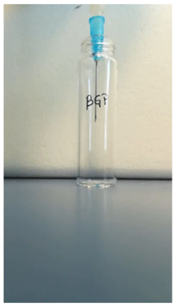

Video S1. Injection test. βGP hydrogel formulation injection through syringes equipped with a 23G needle at r.t.

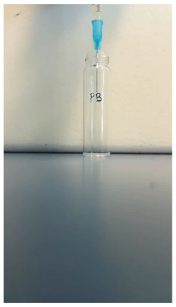

Video S2. Injection test. PB hydrogel formulation injection through syringes equipped with a 23G needle at r.t.

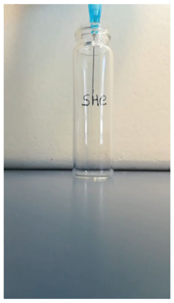

Video S3. Injection test. SHC hydrogel formulation injection through syringes equipped with a 23G needle at r.t.

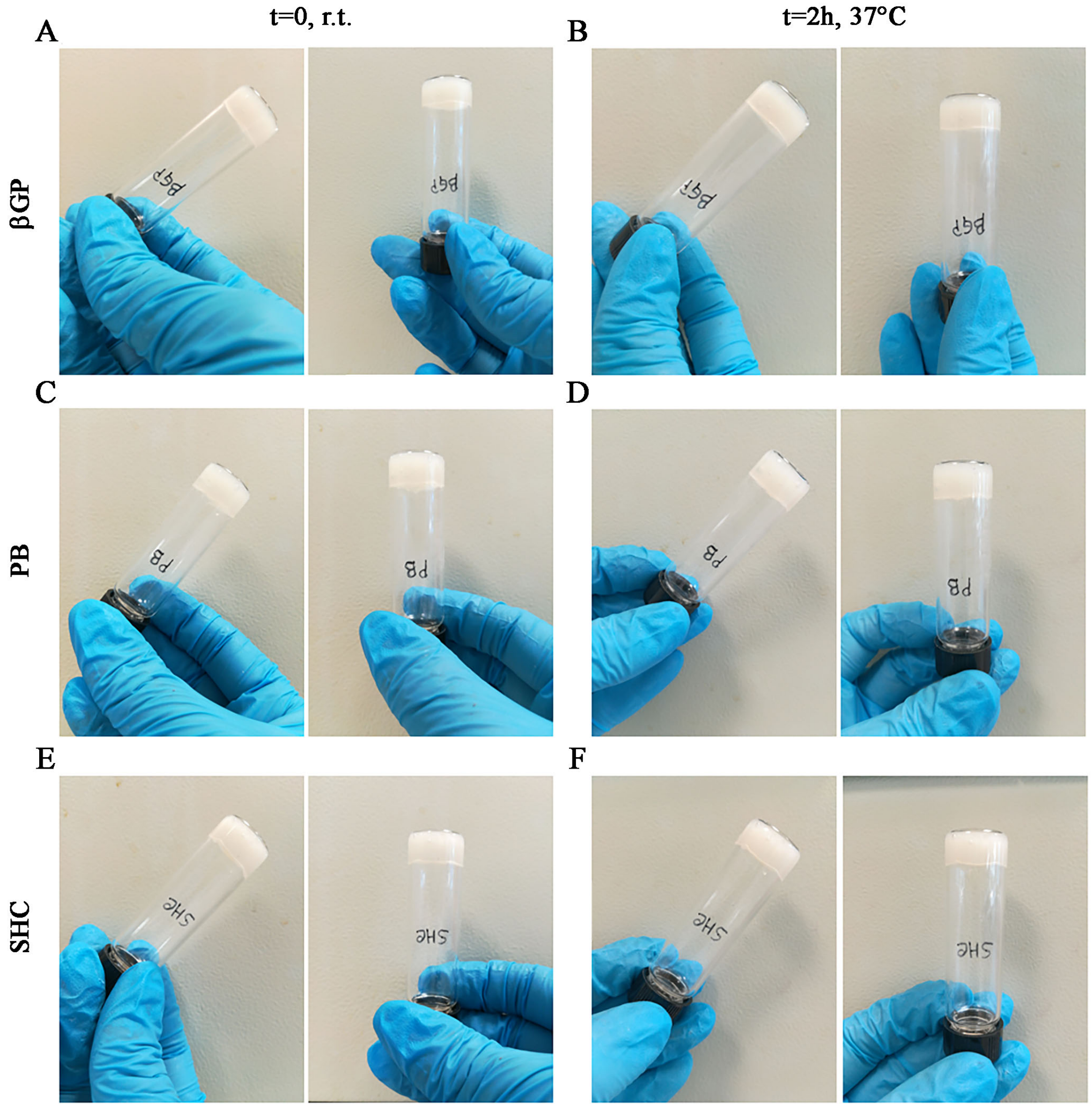

Figure S1. Inversion tube test. The three hydrogel formulations, βGP, PB and SHC, at r.t. after mixing (A, C, E), and after 2h of incubation at 37°C (B, D, F).

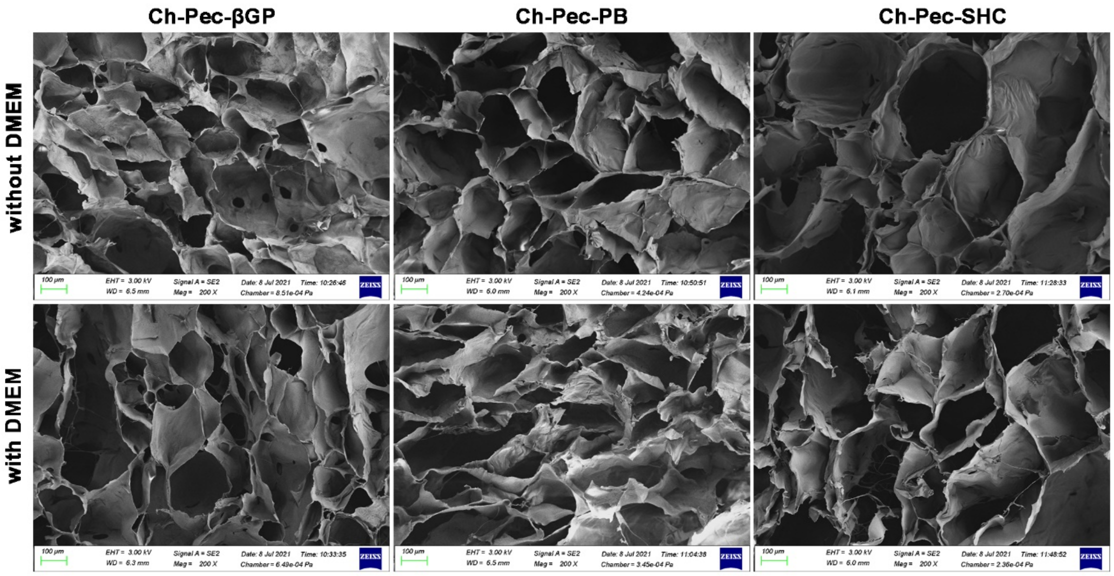

Figure S2. Scanning electron micrographs (SEM). Morphological analysis of hydrogel formulations (βGP-PB-SHC) with and without the addition of DMEM, at magnification 200× (scale bar =100 µm).

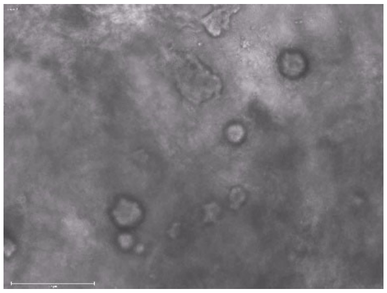

Video S4. Z-stack analysis at 10x of spheroids in Ch-Pec-βGP hydrogels after 21 days of culture.

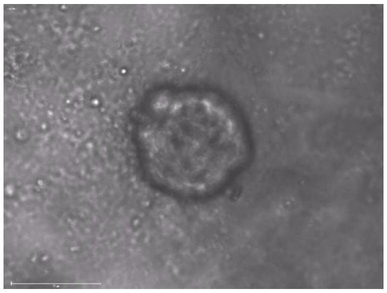

Video S5. Z-stack analysis at 40x of a single spheroid in Ch-Pec-βGP hydrogels after 21 days of culture.
